# Population Genetics of Seaside Sparrow (*Ammodramus maritimus*) Subspecies along the Gulf of Mexico

**DOI:** 10.1371/journal.pone.0112739

**Published:** 2014-11-20

**Authors:** Stefan Woltmann, Philip C. Stouffer, Christine M. Bergeon Burns, Mark S. Woodrey, Mollie F. Cashner, Sabrina S. Taylor

**Affiliations:** 1 School of Renewable Natural Resources, Louisiana State University AgCenter, Baton Rouge, Louisiana, 70803, United States of America; 2 Mississippi State University - Coastal Research and Extension Center, Grand Bay National Estuarine Research Reserve, Moss Point, Mississippi, 39562, United States of America; 3 Department of Biology, Austin Peay State University, Clarksville, Tennessee, 37040, United States of America; Saint Mary's University, United States of America

## Abstract

Seaside Sparrows (*Ammodramus maritimus*) along the Gulf of Mexico are currently recognized as four subspecies, including taxa in Florida (*A. m. juncicola* and *A. m. peninsulae*) and southern Texas (*Ammodramus m. sennetti*), plus a widespread taxon between them (*A. m. fisheri*). We examined population genetic structure of this “Gulf Coast” clade using microsatellite and mtDNA data. Results of Bayesian analyses (Structure, GeneLand) of microsatellite data from nine locations do not entirely align with current subspecific taxonomy. *Ammodramus m. sennetti* from southern Texas is significantly differentiated from all other populations, but we found evidence of an admixture zone with *A. m. fisheri* near Corpus Christi. The two subspecies along the northern Gulf Coast of Florida are significantly differentiated from both *A. m. sennetti* and *A. m. fisheri*, but are not distinct from each other. We found a weak signal of isolation by distance within *A. m. fisheri*, indicating this population is not entirely panmictic throughout its range. Although continued conservation concern is warranted for all populations along the Gulf Coast, *A. m. fisheri* appears to be more secure than the far smaller populations in south Texas and the northern Florida Gulf Coast. In particular, the most genetically distinct populations, those in Texas south of Corpus Christi, occupy unique habitats within a very small geographic range.

## Introduction

Isolated populations of organisms inhabiting patchily distributed habitat often form genetically or morphologically distinct populations that may be recognized as species or subspecies [1]–[3]. From a conservation standpoint, protecting multiple small populations is important to preserving genetic diversity, but can be difficult in practice [4]. The use of subspecies to identify ecologically or morphologically distinct populations has a long history in ornithology [3], [5], but issues regarding diagnostic criteria and the role of subspecies in conservation are continually debated and refined (e.g., [6], [7]). Furthermore, many subspecific designations predate the modern statistical analyses used to evaluate them [8]. In the United States, the Endangered Species Act (ESA) considers “distinct population segments” as potentially eligible for special protections. Thus, it is important to understand whether taxonomically recognized subspecies can reasonably be considered distinct population segments with unique genetic or ecological characteristics [3].

Although the ESA does not specifically define diagnostic criteria [6], population genetic techniques allow a more refined way to identify unique populations and to infer patterns of gene flow among them [3], [5], [6], [9]. Several recent studies have found at least some degree of concordance with population genetic data and subspecific taxonomy (e.g., *Melospiza melodia* [10], *Buteo lineatus* [11], *Myioborus miniatus* [12], *Chondestes grammacus* [13]), but the vast majority of named avian subspecies have not been evaluated using modern techniques and remain in place largely due to “historical inertia” [8].

The Seaside Sparrow (*Ammodramus maritimus*) is largely endemic to - and often abundant in - salt marshes along the Atlantic and Gulf coasts of the United States; seven extant subspecies are currently recognized [14] based on plumage, song, or for some Atlantic birds, mtDNA (reviewed in [15]). The presence of geographic variation in plumage [15] and song [16] provides a basis for inferring limited gene flow among populations, and some subspecies (e.g., *A. m. nigrescens*, *A. m. mirabilis*) have been considered full species in the past [17]. The taxonomic history of the Seaside Sparrow is complex, however, and has been complicated by the difficulty of interpreting subtle plumage characters, within-population plumage variation, and by limited numbers of specimens in fresh basic plumage in museum collections. Furthermore, even obvious differences in plumages among bird populations do not always correlate with genetic differentiation (see e.g., [18]). Based on studies of mtDNA, *Ammodramus m. maritimus*, *A. m. macgillivraii*, *A. m. mirabilis*, *A. m, nigrescens*, and presumably *A. m. pelonota* form an “Atlantic” clade (*A. m. pelonota* has not been analyzed due to lack of specimens; [19], [20]). *Ammodramus m. nigrescens* and *A. m. pelonota* have become extinct since the 1980s, although the distinctness of the latter has been questioned [21]. The “Gulf Coast” clade has a more convoluted taxonomic history [15], [22]–[24], but most recently consists of four subspecies (based on plumage characteristics), from west to east along the Gulf Coast: *A. m. sennetti* (not sampled by Avise and Nelson [20]), *A. m. fisheri, A. m. juncicola*, and *A. m. peninsulae* [14]. The latter two populations are currently listed as Threatened in Florida [25], [26].

Given that at least one subspecies has gone extinct and other named subspecies have quite narrow distributions, a modern genetic analysis is needed to enable an assessment of conservation risk for Gulf Coast Seaside Sparrows. In this paper we: (1) describe population genetic structure of Seaside Sparrows along the Gulf Coast; (2) ask whether genetically distinct populations align with current subspecific taxonomy, and (3) discuss potential conservation risks based on a more refined understanding of the distribution and genetic variation among these populations.

## Methods

Birds were banded and bled under Federal Bird Banding Permit 22648, State Permits LNHP-11-06 and LNHP-12-023 (Louisiana), SPR-1011-351 (Texas), LSSC-11-00096 (Florida), 21553-12-0010 (Laguna Atascosa National Wildlife Refuge), 21540-12-112 (Texas Mid-Coast National Wildlife Refuge Complex), 4164-2011-002 (St. Marks National Wildlife Refuge), 01111210 (Florida Division of Recreation and Parks), and 2011-001 (Rockefeller Wildlife Refuge, Louisiana). This study was carried out in strict accordance with the recommendations in the Guide for the Care and Use of Laboratory Animals of the National Institutes of Health. Protocols were approved by the Institutional Animal Care and Use Committee of the Louisiana State University AgCenter (Permit Numbers: AE2011-04 and A2012-05).

### Study area

**Figure 1:**
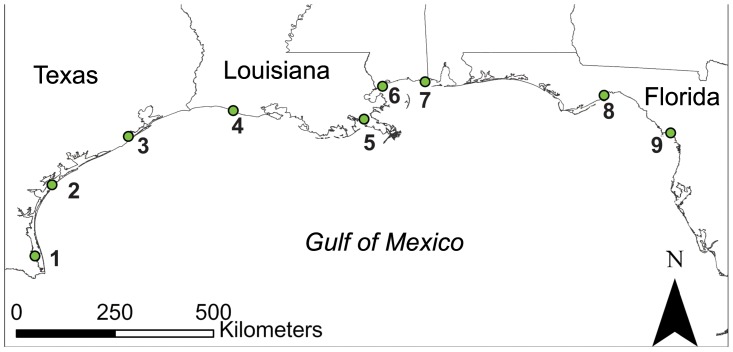
Map of sampling locations along the Gulf of Mexico. See Table 1 for details.

We captured 374 Seaside Sparrows with mist nets at nine locations across the northern coast of the Gulf of Mexico during 2012–2013 (Table 1, Fig. 1). Locations were chosen to include all four currently recognized subspecies, and to sample throughout the range of the subspecies with the broadest geographical distribution (*A. m. fisheri*). Captured birds were banded with numbered aluminum bands; a blood sample was taken from the brachial vein and stored in Queen's lysis buffer [27].

**Table 1:** Sampling localities, presumed subspecies and sample sizes of Seaside Sparrow used in the Gulf-wide analyses of population genetic structure.

Site #	subspecies	Locality	Representative coordinates	N
1	cf. *sennetti*	Cameron Co., Texas	26.34878, −97.37035	25
2	*sennetti* a	Aransas Co., Texas	27.98224, −96.98039	26
3	*fisheri*	Brazoria Co., Texas	29.08142, −95.24178	32
4	*fisheri*	Cameron Par., Louisiana	29.66890, −92.84939	72
5	*fisheri*	Plaquemines Par., Louisiana	29.46040, −89.87547	72
6	*fisheri*	St. Bernard Par., Louisiana, and Hancock Co., Mississippi	30.22652, −89.42580	49
7	*fisheri*	Harrison, Jackson Co., Mississippi	30.33023, −88.45475	36
8	*juncicola*	Wakulla Co., Florida	30.01658, −84.36886	30
9	*peninsulae*	Levy Co., Florida	29.16448, −82.85081	32

a Griscom [62] noted that *sennetti* and *fisheri* from this area may intergrade, even though it is the type locality for *sennetti*.

Subspecific identities based on summary by Robbins [22]. “Site #” refers to localities shown in Fig. 1.

### Laboratory methods

DNA was extracted using Qiagen DNeasy Tissue and Blood kits (Qiagen Ltd.). We quantified extracted DNA concentrations with a NanoDrop 2000 (ThermoScientific, Delaware, USA). DNA from all individuals was amplified for 14 microsatellite loci: Aca01, Aca11 [28], Am02, Am12, Am14, Am18, Am20, Am32 [29], Asμ15 [30], Sosp13 [31], and ZoleC06, ZoleC11, ZoleE11, ZoleF11 [32]. Microsatellite primers were modified with a 19 bp M13 tag [33]. Polymerase chain reactions (PCR) were run in 10 µL volumes and included 3.00 mM MgCl_2_, 0.16 mM dNTPs, 1X buffer (New England Biolabs), 0.04 µM forward primer, 0.38 µM reverse primer, 0.60 µM dye-labeled M13 primer, 0.5 units *Taq* polymerase, and 10 ng DNA. Amplification of some loci was improved with the addition of dimethyl sulfoxide (DMSO; 0.30 µL) and betaine (1.0 µL) to the PCR (loci Asμ15, Aca01, Am08, Am14, Am32, ZoleC06, ZoleC11, ZoleE11, ZoleF11, ZoleH02). The thermal cycling protocol was 94°C for 60s, followed by 35 cycles of 94°C for 30s, annealing temperature (60°C for most; 55°C for Asμ15 and Am 32) for 30s, 72°C for 30s, and a final extension cycle at 72°C for 5 minutes. PCR products were run on an Applied Biosystems 3730 capillary sequencer (Yale University, New Haven, CT, USA), and all genotyping was done by a single individual (SW) using GeneMarker (v. 1.97; SoftGenetics, LLC., State College, Pennsylvania). At least three reference individuals were included on each plate to ensure consistency between runs.

The mitochondrial nicotinamide adenine dinucleotide dehydrogenase subunit 2 (ND2; 1042 bp) was sequenced for 9–12 individuals from sampling locations 1, 2, 3, 6, 8 and 9 (total n = 64; see Fig. 1). ND2 was chosen because it is both relatively long and generally highly variable in birds [34], and mtDNA often provides a different (presumably older) population history signal than microsatellites (e.g., [13], [35]). PCRs were run in 10 µL volumes with the following conditions: 1.50 mM MgCl2, 0.8 mM dNTPs, 1.25 µM each forward (L5215; [36]) and reverse (H6313; [37]) primers, 1X buffer, 2.5 units *Taq* polymerase, and 10 ng DNA. The thermal cycling protocol consisted of 94°C for 30 s followed by 34 cycles of 94°C for 30 s, 50°C for 30 s, 72°C for 60 s, and a final extension step of 72°C for 7 minutes. Sanger single-pass sequencing was performed at Beckman Coulter Laboratories (Danvers, MA, USA). Sequences were aligned in Sequencher (v. 5.1, Gene Codes Corp., Ann Arbor, Michigan) and deposited in GenBank (accession numbers in Table 2).

**Table 2:** ND2 haplotype frequencies for selected population samples of Seaside Sparrow along the coast of the Gulf of Mexico (see Fig. 1 for sampling localities).

	Haplotype^a^												
Site # (N)	1	2	3	4	5	6	7	8	9	10	11	12	13
1 (11)	—	0.55	—	—	—	—	0.46	—	—	—	—	—	—
2 (9)	0.89	0.11	—	—	—	—	—	—	—	—	—	—	—
3 (11)	0.46	0.09	—	—	—	0.09	—	—	0.09	0.09	0.09	0.09	—
5 (10)	0.70	—	—	0.10	—	—	—	0.20	—	—	—	—	—
8 (11)	0.91	—	—	—	0.09	—	—	—	—	—	—	—	—
9 (12)	0.75	—	0.17	—	—	—	—	—	—	—	—	—	0.08

a GenBank accession numbers for the haplotypes: 1: KJ881004, 2: KJ881006, 3: KJ880997, 4: KJ881001, 5: KJ881007, 6: KJ881002, 7: KJ881008, 8: KJ880999, 9: KJ881005, 10: KJ881000, 11: KJ881009, 12: KJ881003, 13: KJ880998.

### Analytical methods

Microsatellite data were checked for evidence of null alleles and potential scoring problems with MicroChecker [38], and within-population HWE and LD with GenePop [39]. Population sample characteristics (allele richness, observed vs expected heterozygosity) were summarized with GenAlEx ([40]; Table 3). Pairwise population differentiation analysis (*F*_ST_; 5,000 permutations to test for significance) and Factorial Correspondence Analysis (FCA; used to visualize degree of difference among population samples) were performed in Genetix [41]. Jost's *D*_ST_ ([42]; as an alternative to *F*_ST_) was estimated in SMOGD [43]. We tested three subsets of the data for isolation by distance (IBD) in IBDWS [44] using 5,000 iterations and *F*_ST_/(1−*F*_ST_) vs log km to explore the influence of some of the samples: (1) all samples; (2) all samples excluding putative *A. m. sennetti* (locations 1 and 2); (3) only samples of putative *A. m. fisheri* (locations 3–7). Effective genetic population size (*N*_e_) was estimated for all nine populations using the LD method with a random-mating model, 0.05 as the lowest allele frequency used, and a jackknife approach to estimating 95% CIs in the program NeEstimator [45]. We chose this LD method because it is widely-used, incorporates a bias-correction for calculating confidence intervals, and requires relatively few assumptions regarding the samples [46]. An AMOVA of ND2 sequences was performed in Arlequin (v. 3.5.1.2; [47]), using genetic distances (*F*_ST_) and pairwise differences (100 permutations) for comparisons. A 95% confidence interval haplotype network was constructed with TCS (v 1.21; [48]).

**Table 3:** Summary population genetic characteristics and estimated effective population size (*N_e_*) of the nine sampled populations.

Site	*N*_a_	*A_r_*	*H*_o_	*H*_e_	*N*_e_	*N*_e_ 95% CI
1	6.071	5.809	0.743	0.695	556	71–∞
2	9.000	8.180	0.836	0.799	283	82–∞
3	10.071	8.693	0.826	0.825	2047	159–∞
4	11.143	8.489	0.837	0.833	∞	451–∞
5	11.357	8.398	0.832	0.824	∞	457–∞
6	10.429	8.238	0.837	0.823	341	149–∞
7	9.214	8.040	0.801	0.815	84	54–164
8	9.071	8.214	0.808	0.808	176	86–5378
9	9.429	8.161	0.830	0.814	857	150–∞

*N*_a_ =  mean number of alleles across all loci, *A_r_* =  mean allelic richness (calculated from per-locus estimates generated with [70]), *H_o_* =  observed heterozygosity, *H*_e_ =  expected heterozygosity. See text for details of the estimation and interpretation of *N*_e_.

We used two Bayesian clustering programs to evaluate the distribution of genetic structure within our study area. For Gulf-wide analyses we used a reduced number of individuals from locations 4 and 5 in order to maintain a more balanced number of samples from each of the nine locations. In the program Structure [v2.3.4; [49], [50] we ran an admixture model (50,000 burn-in, 1,000,000 iterations) with correlated allele frequencies, using sampling locations (locprior), and otherwise default settings We performed 20 runs for each hypothesis of K = 1–11. We processed Structure output with Structure Harvester [51], used clumpp [52] to condense data from multiple runs, and created a graphic of population assignments with distruct [53].

We used GeneLand [54] to explore spatial models for both the entire Gulf-wide dataset and two further subsets of the data. For the Gulf-wide analyses we used a no-admixture and uncorrelated allele frequencies model because IBD can cause problems for some of these algorithms [55]. We performed 10 runs for each hypothesis of *K* = 1–11 (1,000,000 iterations; thinning  = 1,000). Two additional GeneLand analyses were run to further explore data from locations in southern Texas and Florida. To examine an apparent hybrid zone near Corpus Christi, Texas we used a correlated allele model and the admixture model for hybrid zones (populations 1–3; 5 runs for *K* = 1–3; 1,000,000 iterations, thinning  = 1,000). To examine weak differentiation (based on *F*_ST_) of the two named subspecies sampled in Florida (locations 8 and 9) we used a correlated allele model (5 runs for *K* = 1–2; 1,000,000 iterations, thinning  = 1,000). For all GeneLand analyses we set coordinate uncertainty to 0.3 to allow for the possibility of different individuals captured at the same point to be assigned to different populations. Because GeneLand algorithms may produce “ghost” populations (i.e., inferred populations containing no sampled individuals), especially at large spatial scales, we limited our acceptance of GeneLand results to the most parsimonious estimation of *K* that did not include “ghost” populations [54].

To further explore the apparent admixture zone near Corpus Christi, Texas, we used two Bayesian approaches to estimate historic (Migrate-N; [56], [57]) and more recent (BayesAss; [58]) geneflow among the three Texas sampling areas. Migrate-n (v. 3.6.4; [56], [57]) estimates scaled population sizes (θ = 4*N*_e_μ, where μ is the mutation rate) and migration rates (*M* = m/μ, where m =  migration rate). We used a Bayesian framework and a Brownian motion microsatellite model, with a proposed connection matrix that included estimation of all θ and *M* parameters with the exception of migration between locations 1 and 3 (i.e., a ‘stepping stone’ model), as direct migration between these two locations is biologically unreasonable considering the sedentary nature of Seaside Sparrows along the Gulf Coast [14]. Following a series of preliminary runs to explore the possible ranges of priors (uniform distribution), we used the following start and search parameters: both θ and *M* were estimated using *F*_ST_, MCMC runs used one long chain, recording every 100 steps, and visiting 4×10^6^ geneologies.

The program BayesAss (v3.0) estimates recent immigration rates by estimating the proportion of migrant ancestry within population samples and individuals [58]. We followed the recommendations of Wilson and Rannala [58] and the program user's manual by running several trial runs to find search parameters that produced acceptable acceptance rates (0.2–0.6) for estimates of migration rate (M), individual migrant ancestry (A), and inbreeding coefficients (F). We subsequently performed multiple runs with different seeds to insure comparable results were obtained between runs. We ran 10^7^ iterations, with 10^6^ as burn-in, with a sampling interval of 10^3^. Mixing parameters were dM = 0.1, dA = 0.45, and dF = 0.45. We examined the trace outputs of the log-probabilities to insure the MCMC had converged using the program Tracer (v.1.6.0 [59]). Estimates of ancestry for each individual were summarized by adding the estimated ancestry coefficients (source, 1^st^ generation or 2^nd^ generation migrant from each population) to characterize the likelihood of migrant ancestry.

## Results

**Figure 2:**
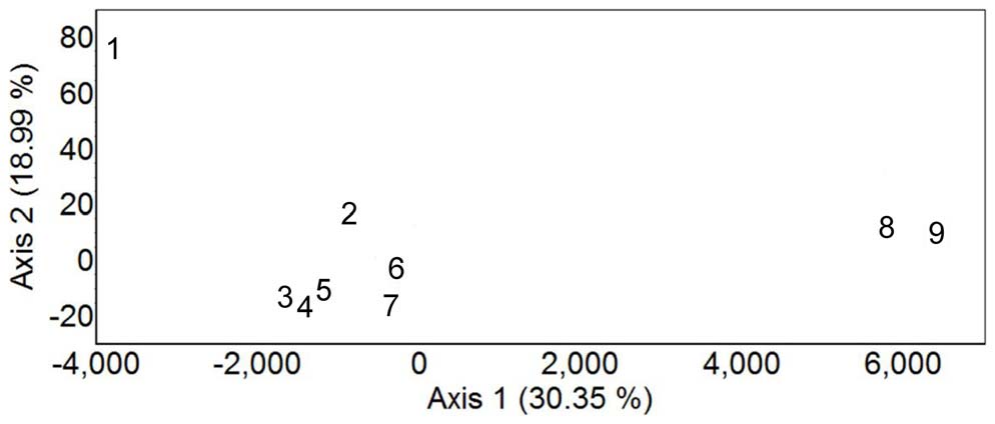
Factorial Correspondence Analysis (FCA) of nine sampled populations of Seaside Sparrow along the Gulf of Mexico. Site numbers are from Fig. 1.

We found no evidence of technical problems relating to microsatellite genotyping, and no evidence of within-location LD or deviation from HWE. The average number of alleles per locus was lowest (6.1) in *A. m. sennetti* from location 1, but similar (range  = 9.0–11.4) among all other locations (Table 3). Estimates of *N*_e_ for the nine population samples were generally high; most confidence intervals included infinity, which Do et al. [45] interpret as a lack of LD signal to use for estimation of *N*_e_ (Table 3). Although this may also be interpreted to mean the population sizes are indeed quite large, high values could also be due to other factors, such as migration and selection [60]. In our study, few populations are likely to be completely isolated, and immigration from adjacent areas seems likely. A majority of pairwise *F*_ST_ values were statistically significant (*P*<0.001); both *F*_ST_ and *D*_est_ were consistently higher in all comparisons involving location 1 (southernmost *A. m. sennetti*), and lowest within samples encompassing *A. m. fisheri* (Table 4). The FCA shows that populations 3–7 are most similar to each other, but that populations 1, and [8+9] are distinct (Fig. 2).

**Table 4:** Pairwise *F*_ST_ (above diagonal) and *D*_est_ (Jost‘s [42]; below diagonal) based on microsatellite data for nine population samples of Seaside Sparrow along the coast of the Gulf of Mexico.

	Site 1	Site 2	Site 3	Site 4	Site 5	Site 6	Site 7	Site 8	Site 9
Site 1	—	0.0438	0.0544	0.0492	0.0504	0.0547	0.0594	0.0886	0.0796
Site 2	0.0647	—	0.0183	0.0169	0.0230	0.0218	0.0227	0.0338	0.0294
Site 3	0.1156	0.0243	—	(-0.0011)	(0.0047)	(0.0053)	(0.0083)	0.0264	0.0204
Site 4	0.1157	0.0265	0.0000	—	(0.0007)	(0.0013)	0.0072	0.0228	0.0201
Site 5	0.1383	0.0348	0.0047	0.0000	—	(-0.0003)	(0.0030)	0.0198	0.0178
Site 6	0.1227	0.0316	0.0081	0.0001	0.0000	—	(0.0033)	0.0168	0.0104
Site 7	0.1552	0.0477	0.0193	0.0140	0.0019	0.0018	—	0.0224	0.0158
Site 8	0.2190	0.0880	0.0802	0.0777	0.0565	0.0368	0.0556	—	(0.0085)
Site 9	0.2143	0.0758	0.0591	0.0620	0.0427	0.0251	0.0378	0.0086	—

*F*_ST_ values in parentheses are not statistically different from zero (*P*>0.001). Negative values of *D*_est_ are reported as zero. See Fig. 1 for sampling locations.

There was a significant pattern of IBD across all nine locations within the microsatellite data (slope  = 0.066, Z = 2.5737, *r* = 0.5346, *P* = 0.0004; Fig. S1A in File S1). Excluding both population samples of putative *A. m. sennetti*, a pattern of IBD was still evident from central Texas to Florida (locations 3–9; slope  = 0.073, Z = 0.9224, *r* = 0.6179, *P* = 0.0036; Fig S1B in File S1). Considering only samples of *A. m. fisheri*, IBD was significant, but relatively weak (locations 3–7; slope  = 0.010, Z = 0.0835, *r* = 0.6318, *P* = 0.0174; Fig. S1C in File S1). IBD using the ND2 data was not significant (slope  = 0.436, Z = 5.1947, *r* = 0.1713, *P* = 0.1630; Fig S1D in File S1).

All six locations sampled for ND2 contained at least one unique haplotype (Table 2, Fig. S2 in File S1). Location 1 was unique in missing the most frequent haplotype found in the other five locations, and also in having only two haplotypes in similar proportions. Overall population structure was significant for the six locations considered (φ_ST_ = 0.1833, *P*<0.0001), and was also evident when excluding location 1 as an outlier with a very different distribution of haplotype frequencies (φ_ST_ = 0.0219, *P* = 0.0585). All pairwise comparisons of φ_ST_ involving location 1 were significant (*P* = 0.0180 for Pop 1 vs Pop 2; all other *P*<0.0001); no other pairwise comparisons were significant (all *P*>0.0528).

### Bayesian inference of K

**Figure 3:**
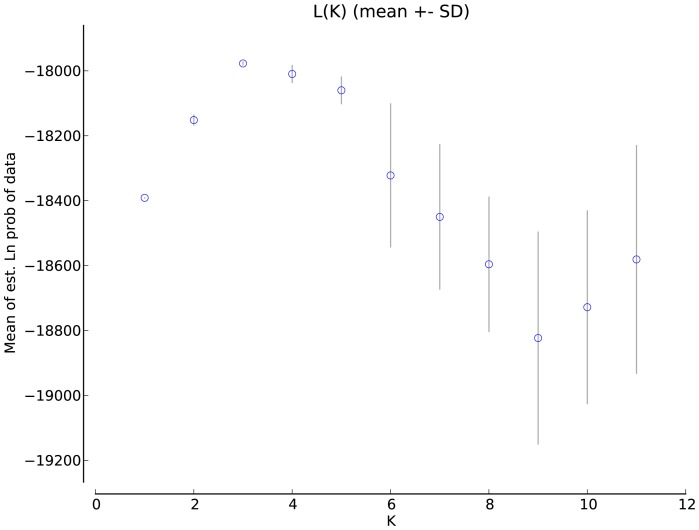
Mean estimated ln probabilities (± SD) of the data for each hypothesis of *K* = 1–11 derived in the program Structure.

**Figure 4:**
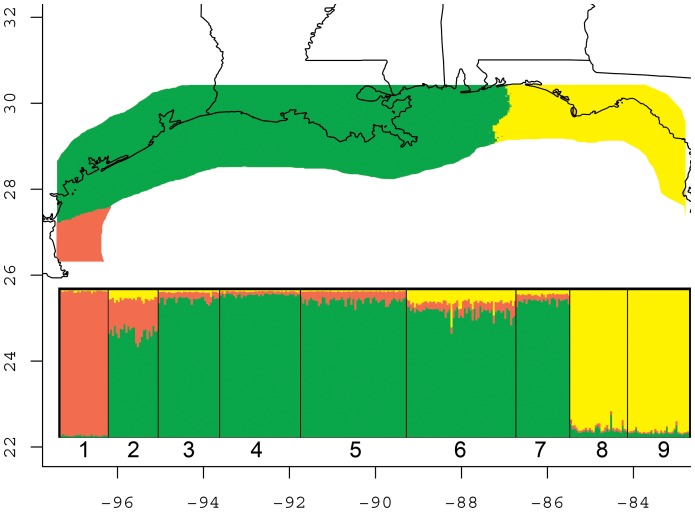
Graphical output of GeneLand (map) and Structure (inset) analyses for best *K* = 3 along the coast of the Gulf of Mexico. Numbers below Structure output correspond to the nine sampled populations of Seaside Sparrow (see Fig. 1). GeneLand output has been cropped to remove much of the area (Gulf of Mexico) not occupied by Seaside Sparrows. Note that no birds were sampled from the western Florida panhandle, and no population assignment for birds in this area is possible based on our data. Axes (latitude and longitude) are only relevant to GeneLand output.

Inspection of plots of summary statistics provided by Structure indicated a sufficient burn-in length and number of post-burn-in iterations. For the Gulf-wide analyses, Structure indicated the best support for *K* = 3, based both on evaluation of mean estimated ln probabilities for each hypothesis (Figs. 3 and 4), and also using the method of Evanno et al. [61] (not shown). The hypothesis of *K* = 4 had the next best support, and differed only in treating location 2 (Aransas Co., TX) as a separate population. GeneLand found a best estimate of *K* = 3 that corresponded well with Structure output (Fig. 4), and the next best GeneLand model also suggested *K* = 4, also treating location 2 separately (not shown).

The admixture analysis in GeneLand of locations 1–3 suggests that birds sampled in Aransas Co. have mixed ancestry of *A. m. sennetti* from further south and *A. m. fisheri* from further north (not shown), and this is congruent with the broader-scale output of the Structure analysis (see Fig. 4, location 2). A correlated allele model in GeneLand consistently recovered a single population among the two samples from Florida (populations 8 and 9), consistent with the low levels of differentiation observed via *F*_ST_ and *D*_est_.

**Figure 5:**
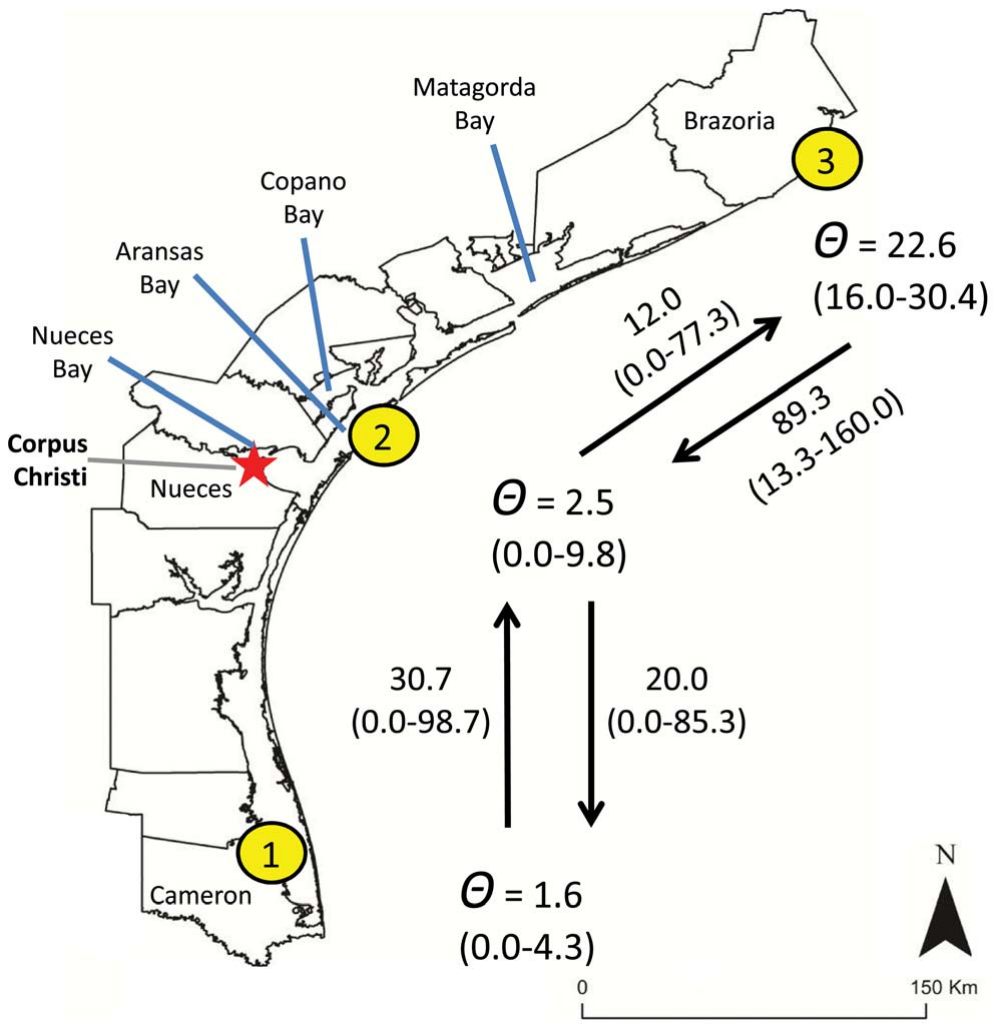
Detailed map of southern Texas showing (1) sampling locations for sites 1–3, (2) bays around Corpus Christi relevant to our discussion of *Ammodramus maritimus* cf. *sennetti*, and (3) estimation of scaled effective genetic population sizes (θ) and directional migration rates (±95% CIs) estimated with the program migrate-n.

**Figure 6:**
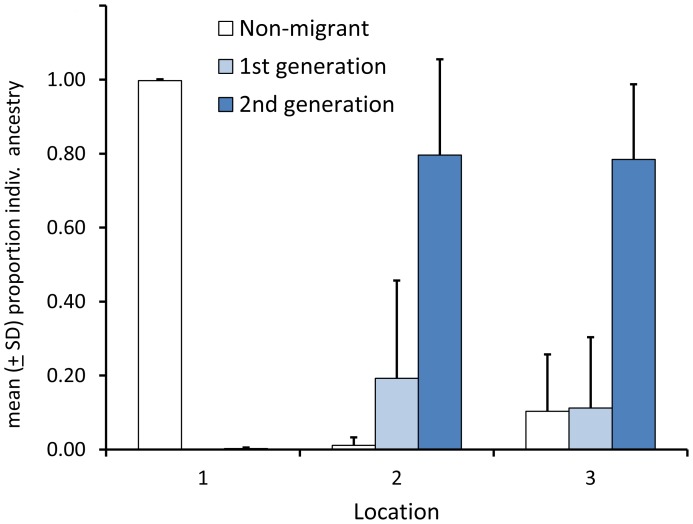
Mean (± SD) proportion of migrant ancestry of individuals within three samples of Seaside Sparrows in Texas, as estimated by the program BayesAss. Note that “2^nd^ generation” estimates are best interpreted as “2^nd^ generation or greater” (see text for additional details). Location numbers are as in Fig. 1.

The Migrate-N analysis of the admixture zone between *A. m. sennetti* and *A. m. fisheri* suggests that our samples from Aransas Co., Texas have historically experienced unequal gene flow from populations to the north and south. Migration rates (*M*) between populations 1 and 2 are roughly similar in each direction. Estimated migration rates between populations and 2 and 3 are asymmetric, with population 3 contributing a greater number of migrants into population 2 (Fig. 5). Recent gene flow among the three Texas sampling locations (as estimated in the program BayesAss) also appears asymmetric, in that, in contrast to populations 2 and 3, population 1 has not received many recent migrants (Table 5), and a large proportion (ca. 0.8) of individuals in populations 2 and 3 have estimated ancestry coefficients consistent with 2^nd^ generation (or greater) migrant (Fig. 6), as would be expected when gene flow is high [58].

**Table 5:** Estimated proportion of self-recruits (m; i.e., the proportion of individuals estimated to have originated within the population) in each of the three Texas population samples, as estimated by the program BayesAss.

Location	M	sd	95% credible set		
1	0.975	0.017	0.942	-	1.000
2	0.682	0.014	0.655	-	0.709
3	0.708	0.032	0.645	-	0.771

The 95% credible set is calculated as the mean ± SD*1.96. Location numbers are as in Fig. 1.

## Discussion

At the broadest geographic scale, our microsatellite and mtDNA analyses show that genetically differentiated Seaside Sparrow populations along the coast of the Gulf of Mexico are not entirely consistent with current subspecific taxonomy. Our microsatellite data are consistent with the recognition of three populations: *A. m.* cf. *sennetti* (south of Corpus Christi, TX – see below), *A. m. fisheri*, and a combined group including *A. m. juncicola* and *A. m. peninsuale*. *Ammodramus m.* cf. *sennetti* from our southernmost site in Texas (location 1) are significantly diverged from all other population samples, including the apparent hybrid *A. m. sennetti* × *fisheri* population in Aransas Co., Texas (location 2). The two Florida subspecies we sampled (*A. m. juncicola* and *peninsulae*) are not significantly differentiated from each other at the 14 loci we sampled, and form a single cluster diverged from the other subspecies in Gulf-wide Bayesian analyses. The subspecies with the largest geographic range (*fisheri*) exhibits relatively weak geographic structuring and IBD throughout its range from the mid-Texas coast to eastern Mississippi.

That Seaside Sparrows sampled near Corpus Christi, Texas (location 2) show genetic evidence of admixture is concordant with Griscom's [62] discussion regarding lighter and darker plumages of Seaside Sparrows between Nueces Bay and Matagorda Bay (a distance of ca. 130 km; see Fig. 5): “the series is sufficiently large, so that one extreme passes into the other extreme by a perfect gradation” (p. 106). Saltmarsh habitat dominated by *Spartina* and *Juncus* (i.e., the usual habitat of Seaside Sparrows along the Gulf Coast) reaches its southern limit in Texas [63], and thus from an ecological perspective, the admixture zone of *A. m. sennetti* and *fisheri* aligns with a shift to different coastal plant communities from north to south. The birds we sampled in Cameron Co. (location 1) were found in habitat dominated by *Borrichia* and *Batis* – a plant community quite unlike any other Seaside Sparrow habitat. This transition in plant communities in the Corpus Christi area is also concordant with Webb's [64] delineation of a “Rio Grande” biogeographic zone for mammals. From a conservation standpoint, the existence of a morphologically, ecologically, and genetically distinct population restricted to a coastal area from Cameron Co. north to somewhere south of Corpus Christi should be of concern. The coastline distance between locations 1 and 2 is ca 200 km, and we do not know (1) where within that range the population genetic characteristics shift to those of *A. m.* cf. *sennetti* from location 1.; and (2) how much of this part of the Texas coast is occupied by Seaside Sparrows. Our data suggest that the geographic range of this taxon is smaller than that most recently described by Oberholser [65] as between Refugio and Cameron counties. Additional data are needed to better characterize and delineate the apparent admixture zone between Cameron Co. and Brazoria Co, and to explore whether the Aransas population (population 2) might be better considered an independent taxon.

Based on the foregoing, we suggest that a taxonomic reassessment of “*A. m. sennetti*” is needed. The type locality of *sennetti* is Corpus Christi [59], ca 40 km southwest of our sampling location 2 (Aransas Bay). Griscom [62] was either unaware of, or did not accept populations south of Corpus Christi, and restricted the range of *A. m. sennetti* to Nueces and Copano Bays (see Fig. 5). Microsatellite allele and ND2 haplotype frequencies of the birds we sampled in this area (location 2; <20 km east of Copano Bay) are more similar to allele frequencies found throughout the range of *A. m. fisheri* than to frequencies found in *A. m.* cf. *sennetti* from location 1 to the south. This prompts the question of whether the type specimen of *A. m. sennetti* is in fact of mixed ancestry, or whether the range of *A. m. fisheri* has expanded southward since the late 1800s, when *A. m. sennetti* was described. In light of this, until the type specimen of *A. m. sennetti* is analyzed from a genetic standpoint, we refer to the birds from Cameron Co., Texas (location 1) as *A. m.* cf. *sennetti*.

The distinction between *A. m. juncicola* and *A. m. peninsulae* has long been debated, generally because plumage variation between the two is slight relative to plumage variation within each area [15]. Pairwise estimates of *F*_ST_ and *D*_ST_ for these two populations (8 and 9; ca 170 km apart) are marginally greater than several estimates for populations of *A. m. fisheri* separated by greater geographic distances. GeneLand analyses - which are explicitly designed to assess fine-scale genetic structure - failed to distinguish between the two Florida populations we sampled. Based on our relatively limited genetic sampling, however, we are hesitant to make any taxonomic suggestions regarding these taxa, and genome-wide sampling, for example, could discover important differences between the populations (see [66]). Nonetheless, even as a combined group, they are genetically (this study) and ecologically [16], [24], [67] distinct from other populations of Seaside Sparrow. Moreover, marsh habitat is restricted in the region to an estimated maximum of 275 and 376 km^2^ for *A. m. juncicola* and *peninsulae*, respectively [25], [26]. Within this range, occurrence of both taxa is often spotty, even in seemingly suitable habitat ([24], SW *pers. obs.*), and additional data are needed to better understand the size, distribution, and demography of these populations. The limited extent of salt marsh within the geographic range of these taxa suggests that marsh loss in this region could decimate these populations [25], [26].

We note that the western panhandle of Florida (Escambia Co. to approximately Bay Co.) remains a gap in our knowledge, at least partially because the limited amount of saltmarsh habitat means that there are very few Seaside Sparrows in the area [23], [24]. Although some published range maps depict this area as within the range of *A. m. juncicola* (e.g., [14], [20], [68]), this view may have begun as a misinterpretation of the range described (albeit somewhat ambiguously) by Griscom and Nichols [69]. This interpretation was abandoned by Robbins [22], and we consider the descriptions of the ranges of both *A. m. juncicola* and *A. m. peninsulae* by the Florida Fish and Wildlife Conservation Commission [25], [26] as the most reasonable interpretation of the original descriptions for those taxa [23], [69]. We caution against interpreting our Fig. 4 as supporting the existence of *A. m. juncicola* and/or *A. m. peninsulae* in the western Florida panhandle because in this case it is a matter of the software (GeneLand) “filling in the gaps.” Analyses of samples from the western Florida panhandle are needed to understand the population genetic affinities of these birds.

In contrast to the case of *A. m. sennetti* and the situation in western Florida, the case of *A. m. fisheri* seems relatively straightforward from Brazoria Co., Texas eastward. Population genetic characteristics of Seaside Sparrows sampled from Brazoria Co. to Jackson Co., Mississippi (populations 3–7) were found to be relatively homogenous and essentially panmictic. This broad distribution and large (combined) population size suggests this taxon is of relatively low conservation concern at this time.

## Summary

In light of recent extinctions (*A. m. nigrescens* and *A. m. pelonota*) and current Federal Endangered status (*A. m. mirabilis*) of taxa within the Seaside Sparrow, our results support the consideration of Seaside Sparrows along the northern coast of the Gulf of Mexico as comprising three “distinct population segments:” (1) *Ammodramus m.* cf. *sennetti*, (2) *A. m. fisheri*, which ranges from at least Brazoria Co., TX, east to Jackson Co., MS, and (3) a combined *A. m. juncicola* + *A. m. peninsulae* group which ranges from Bay Co. south to Pasco Co., FL. Additional data are needed to better understand the situation in Texas south of Brazoria Co., but at the very least our data indicate that a highly distinct taxon (*A. m.* cf. *sennetti*) occurs south of about Corpus Christi. This distinct and isolated taxon may be in need of additional protection and management.

## Supporting Information

File S1 **Figures S1 (showing plots of isolation by distance for microsatellite and mitochondria genotype data) and S2 (showing and ND2 haplotype network).**
(DOCX)
