# Supplementary material for: Population Genetics of Seaside Sparrow (Ammodramus maritimus) Subspecies along the Gulf of Mexico
Source: PLoS One. 2014 Nov 20;9(11):e112739. doi: 10.1371/journal.pone.0112739 (PMC4239047; doi:10.1371/journal.pone.0112739)
Supplement: File S1 — Figures S1 (showing plots of isolation by distance for microsatellite and mitochondria genotype data) and S2 (showing and ND2 haplotype network). (DOCX) [file pone.0112739.s001.docx]

Population Genetics of Seaside Sparrow (*Ammodramus maritimus*) Subspecies Along the Gulf of Mexico.

Stefan Woltmann^1,4,5^ , Philip C Stouffer^1^ , Christine M. Bergeon Burns^1^ , Mark S. Woodrey^2^ , Mollie F. Cashner^3^ , and Sabrina S. Taylor^1^

**SUPPORTING INFORMATION**

Seaside Sparrow microsatellite data can be found at [**http://dx.doi.org/10.7266/N7FT8HZH**](http://dx.doi.org/10.7266/N7FT8HZH)**.**


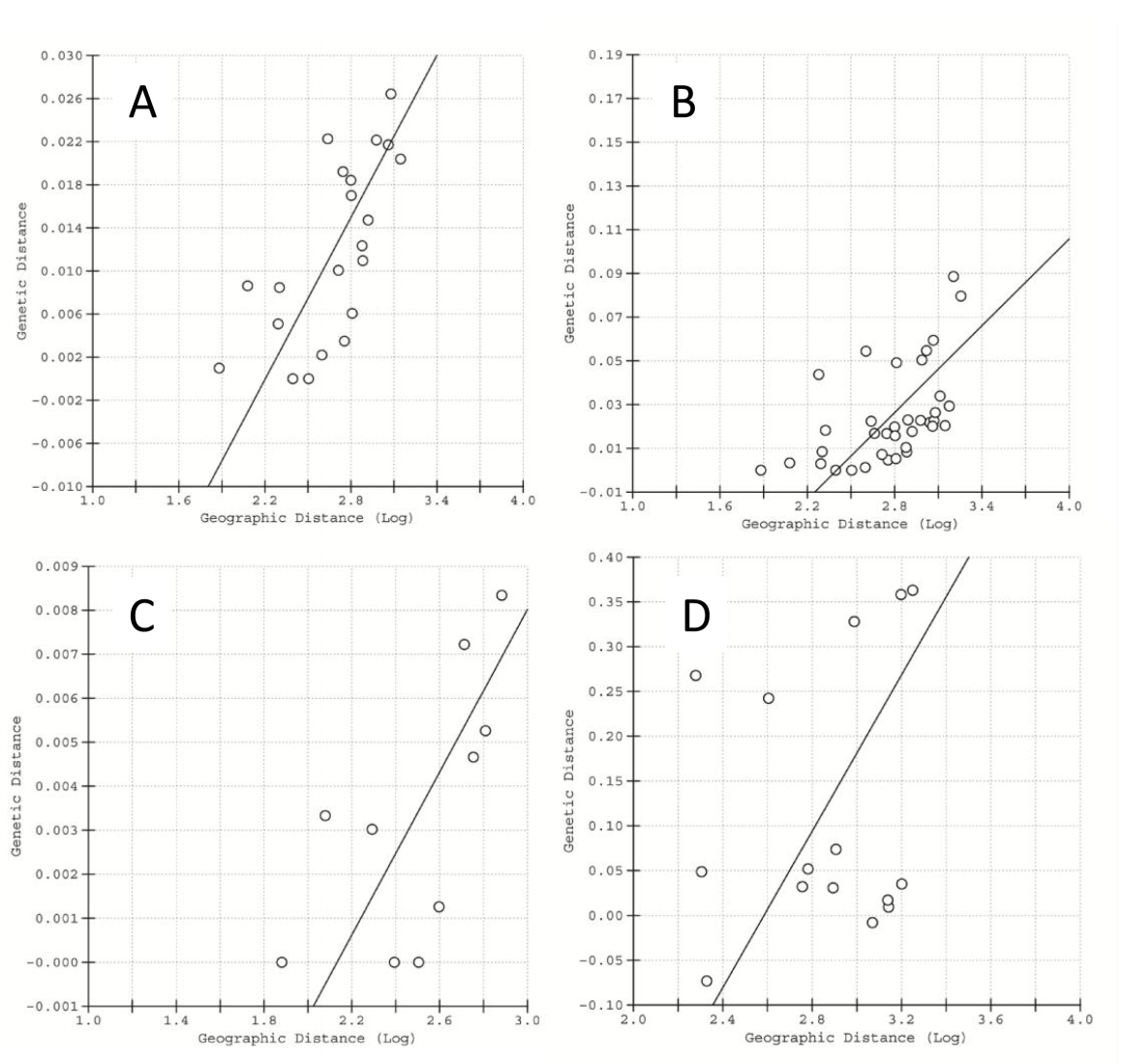


Fig. S1. Plots of genetic distance (*F*_ST_/(1 – *F*_ST_) vs geographic distance (log km) of Seaside Sparrows sampled along the coast of the Gulf of Mexico, as generated by IBDWS [44]. A – all samples (microsatellites); B – populations 3 – 9 (excluding putative *sennetti*; microsatellites); C – only *Ammodramus m. fisheri* (locations 3 – 7; microsatellites); D – ND2 for 6 sampled locations. Note changes to y axis among the plots. See text for summary statistics.


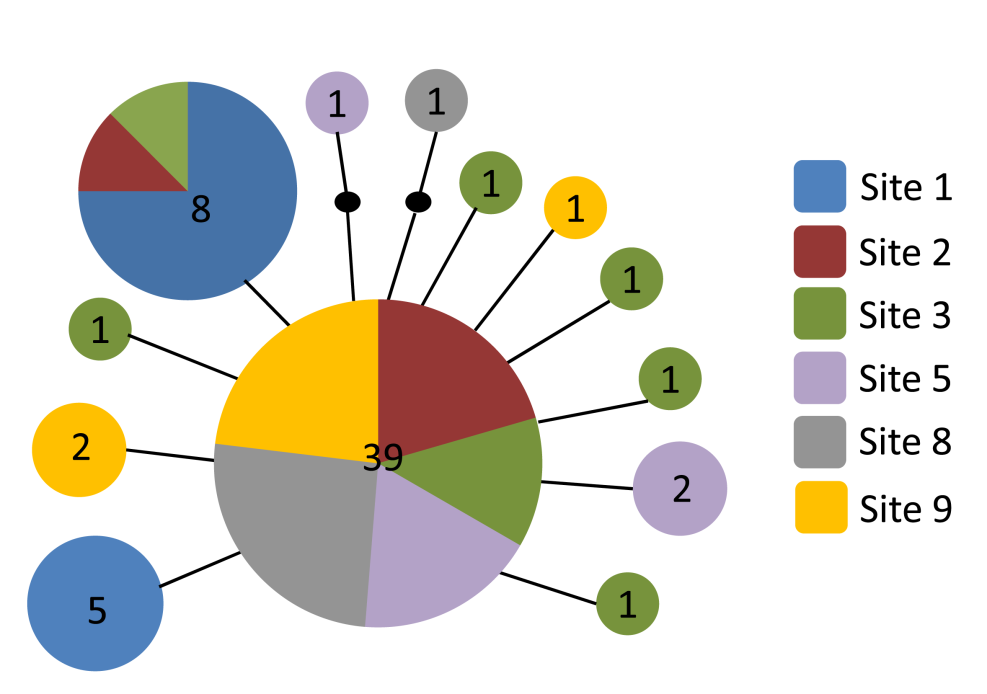


Fig. S2. ND2 haplotype frequencies among six Seaside Sparrow sampling locations along the coast of the Gulf of Mexico. Each circle represents a unique haplotype; numbers within circles represent the number of individuals with that haplotype, color-coded by sampling site.
